# Supplementary material for: Determinants of species-specific utilization of ACE2 by human and animal coronaviruses
Source: Commun Biol. 2023 Oct 17;6:1051. doi: 10.1038/s42003-023-05436-3 (PMC10582019; doi:10.1038/s42003-023-05436-3)
Supplement: Supplementary file 2 — Supplemental Information [file 42003_2023_5436_MOESM2_ESM.pdf]

Qingxing Wang[1], Sabrina Noettger[1], Qinya Xie[1], Chiara Pastorio[1], Alina Seidel[1],
Janis A. Müller[1#], Christoph Jung[2,3,4], Timo Jacob[2,3,4], Konstantin M.J. Sparrer[1], Fabian Zech[1],
and Frank Kirchhoff[1*]

[1]Institute of Molecular Virology, Ulm University Medical Center, 89081 Ulm, Germany;
[2]Institute of Electrochemistry, Ulm University, 89081 Ulm, Germany; [3]Helmholtz-Institute Ulm (HIU) Electrochemical Energy Storage, 89081 Ulm, Germany; [4]Karlsruhe Institute of Technology (KIT), 76021 Karlsruhe, Germany

# Current address: Institute of Virology, Philipps University Marburg, 35043 Marburg, Germany

* Address Correspondence to: Frank.Kirchhoff@uni-ulm.de

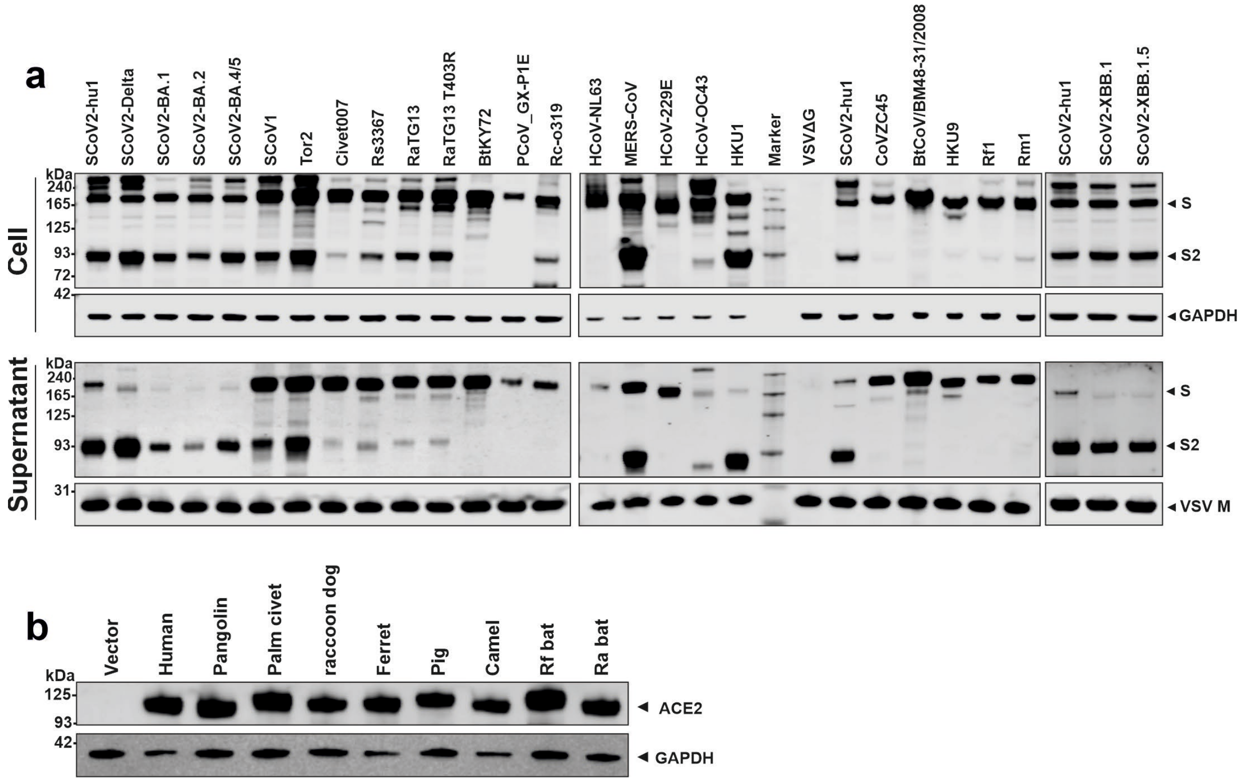

Supplementary Figure 1. Expression of Spike proteins and ACE2 receptors analyzed. a, Immunoblots of whole cells lysates and supernatants of HEK293T cells transfected with vectors expressing the indicated S proteins. Blots were stained with anti-strep tag, anti-V5 tag, anti-GAPDH, and anti-VSV-M. Upper blots of cellular extracts (Cell) and the VSVpp-containing culture supernatant (Supernatant) were derived from different membranes. **b**, Western blot analyses of extracts of HEK293T cells transfected with expression constructs for the indicated ACE2 orthologs or an empty control vector. Uncropped blots are shown in Supplementary Figure 9.

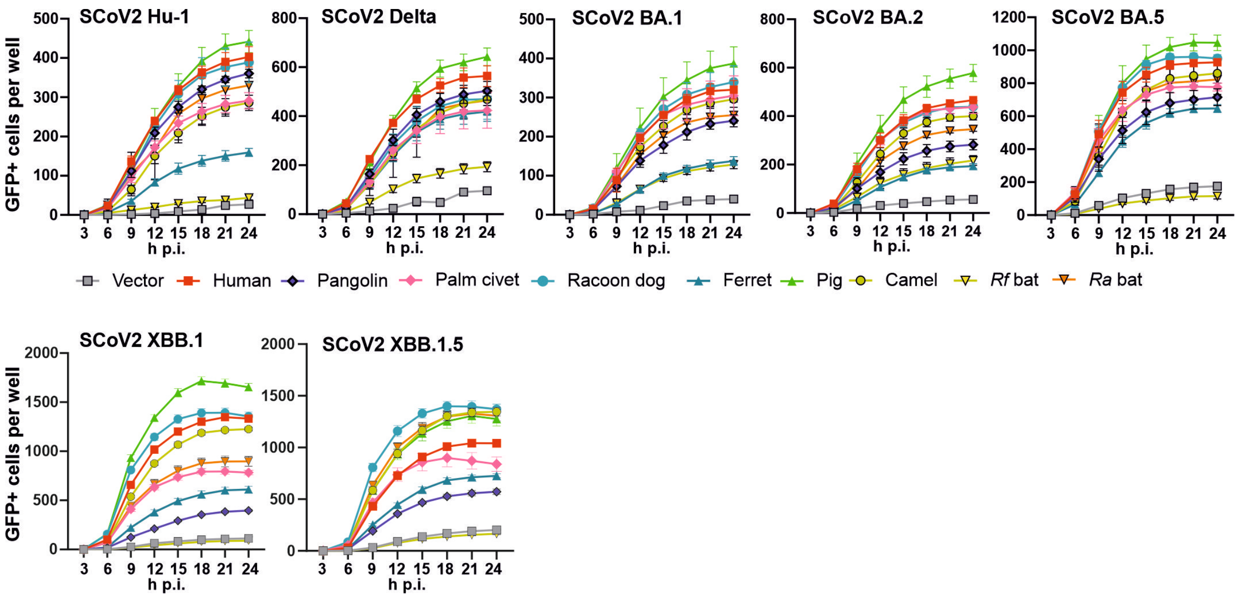

Supplementary Figure 2. Utilization of different ACE2 orthologs by the S proteins of SARS-CoV-2 variants. Infection kinetics of HEK293T cells expressing ACE2 receptors from the indicated species after exposure to VSVpp containing the S proteins of SARS-CoV-2 variants. Infected GFP+ cells were automatically quantified over a period of 24 h. Bars in all panels represent the mean of four experiments (± SEM).

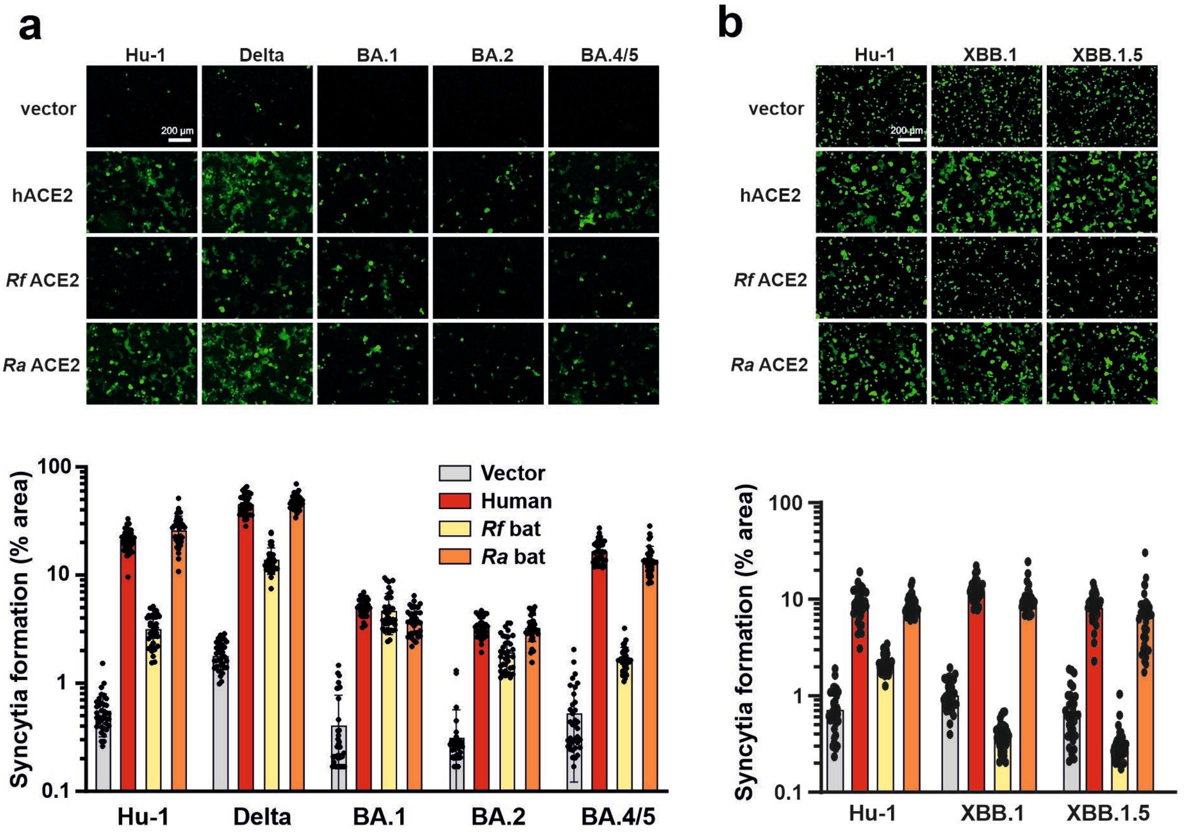

Supplementary Figure 3. BA.5 S does not allow *Rf* ACE2 dependent cell fusion. Left, Exemplary fluorescence microscopy images of HEK293T cells expressing SARS-CoV-2 S protein and the indicated ACE2 orthologs (left). Scale bar, 200 µm. The right panel shows the automatic quantification of syncytia formation by calculating the GFP positive areas. Bars represent the mean of three independent experiments (±SEM).

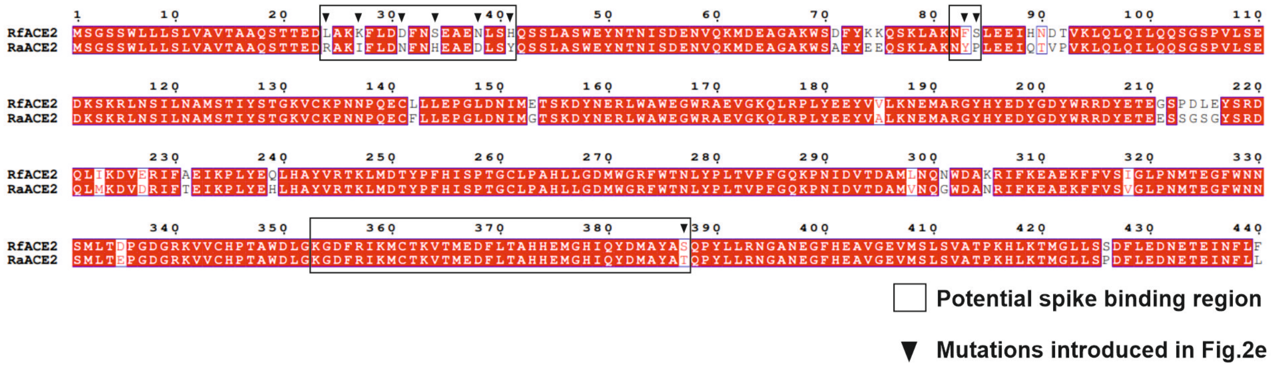

Supplementary Figure 4. Mutations introduced into *Rf* ACE2. Conserved areas are indicated in red. Number give amino acid positions in the *Rf* and *Ra* ACE2 sequence.

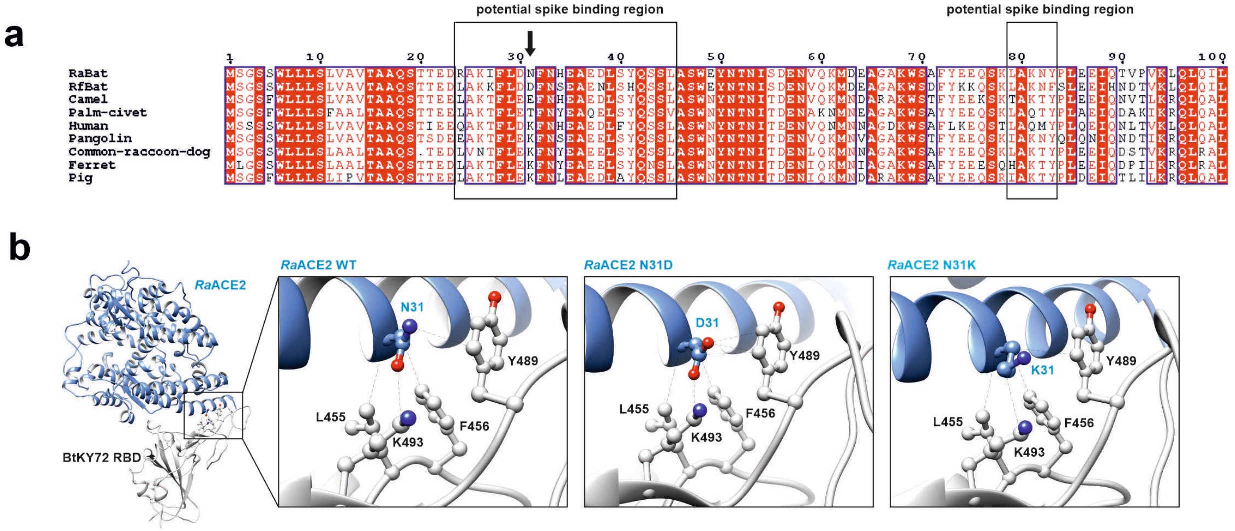

Supplementary Figure 5. Potential determinants of species-Specificity of ACE2 receptor function. a, Alignment of ACE2 sequences analyzed. Residues that are generally conserved are shown in white letters with red background; red letters indicate residues found in most sequences and black letters variable residues. Conserved areas are further indicated by blue boxes. **b.** Schematic diagram of BtKY72 RBD with the *Ra* ACE2. Potential van der Waals interactions of BtKY72 RBD with the *Ra* ACE2 wide-type, N31D or N31K are indicated by dash back lines.

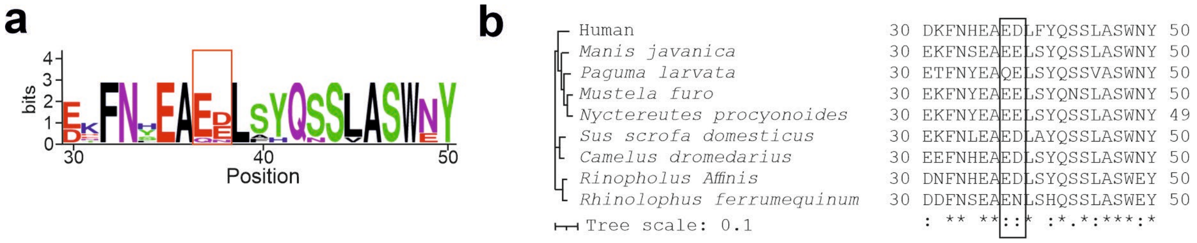

Supplementary Figure 6. Conservation of acid amino acids at positions 37 and 38 of the ACE2 orthologs analyzed. a, Sequence logo of the alignment of ACE2 sequences between sequence positions 30 and 50. Positions 37 and 38 are highlighted by a red box. **b,** Alignment of amino acid residues 30 to 50 of ACE2 sequences analyzed.

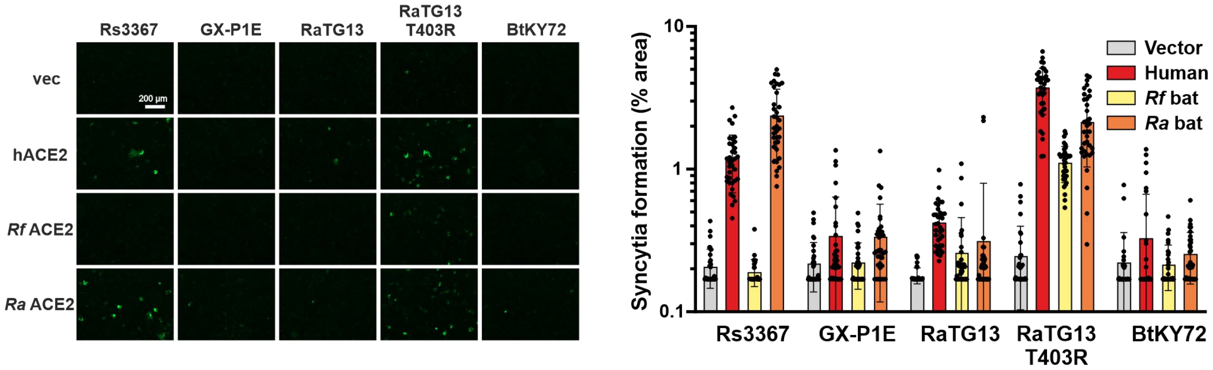

Supplementary Figure 7. Species-specific ability of bat CoV S proteins for ACE2-mediated cell-to-cell fusion. Fluorescence microscopy images of HEK293T cells expressing S proteins of bat CoVs and the indicated ACE2 orthologs (left). Scale bar, 200 µm. The right panel shows shows the automatic quantification of syncytia formation by calculating the GFP positive areas. Bars represent the mean of three independent experiments (±SEM). Statistical significance was tested by two-tailed Student's t test with Welch's correction. *p < 0.05; **p < 0.01; ***p < 0.001.

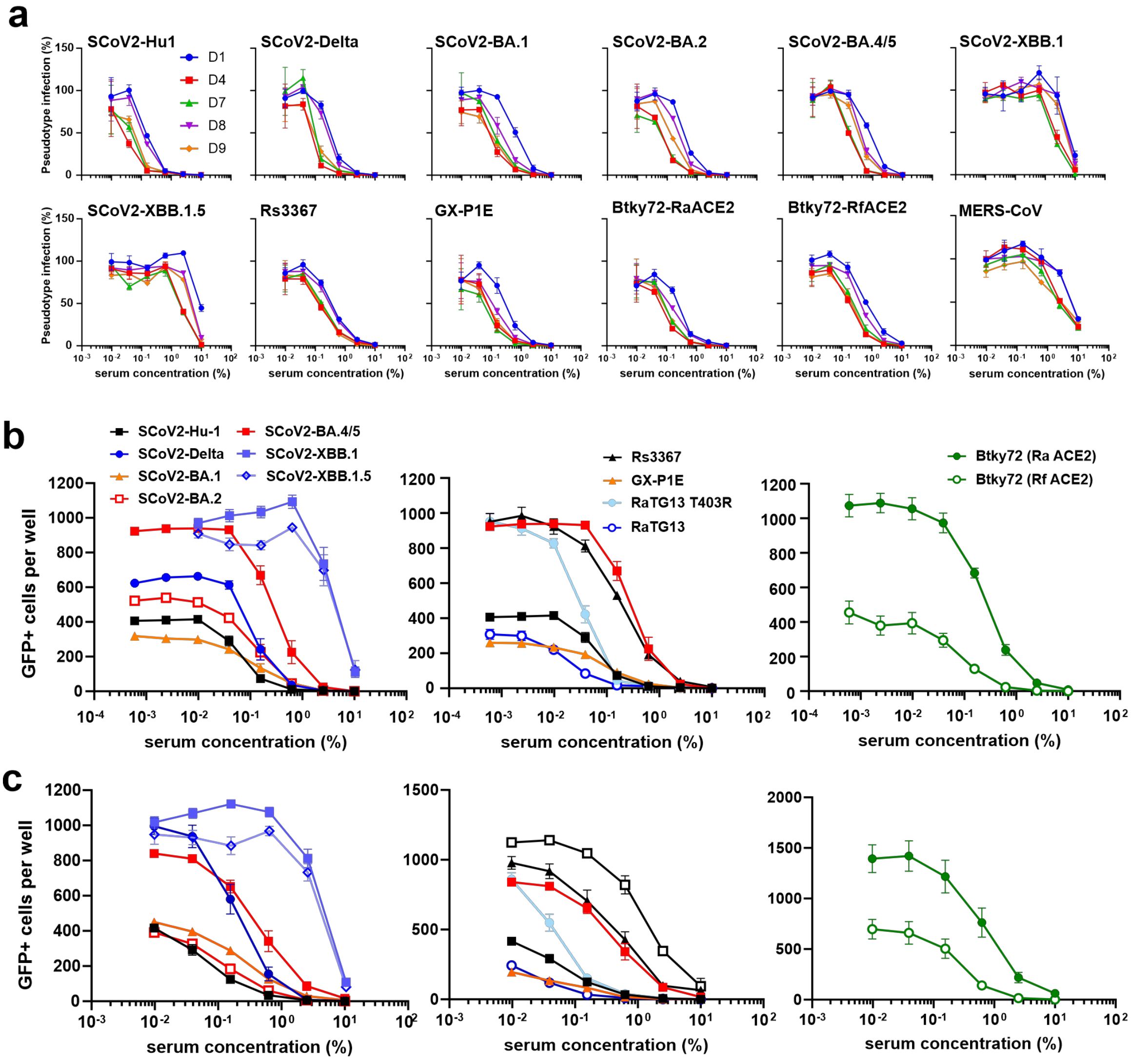

Supplementary Figure 8. Neutralization of VSVpp infection mediated by S proteins of human and bat CoVs. a, Neutralization of VSVΔG-GFP pseudotyped with the indicated S proteins by sera from five AZ/2xBNT162b2 vaccinated individuals. Lines represent the mean of at least two biological replicates. **b, c**, Infection of HEK293T cells expressing human or bat (right panels) ACE2 receptors by VSVpp carrying the S proteins of the indicated human and bat CoVs in the presence sera obtained from five AZ/BNT/BNT (b) or five 3xBNT (c) vaccinated individuals. Shown are total numbers of VSVpp infected (GFP+) cells in the presence of the indicated concentrations of sera. Shown are mean values obtained for the five sera, each tested in two technical replicates.

Supplementary Figure 9

Fig.2b

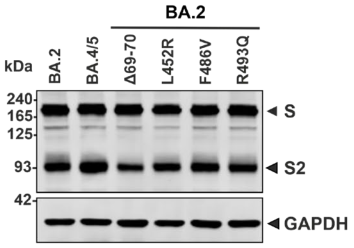

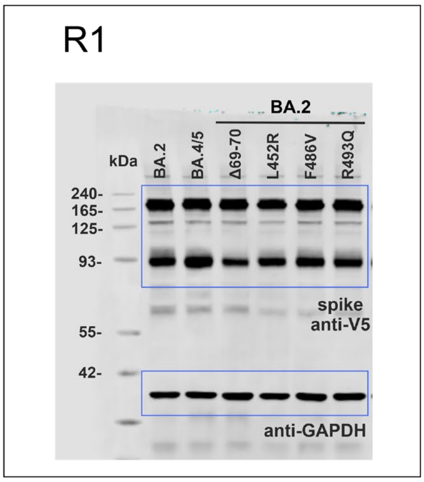

Above single membrane staining

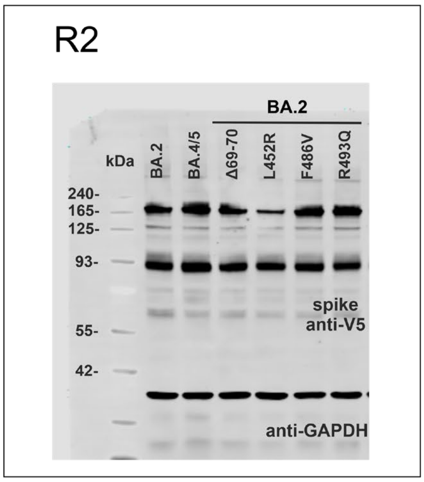

Above single membrane staining

Fig.2e

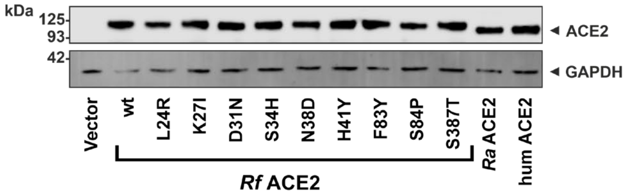

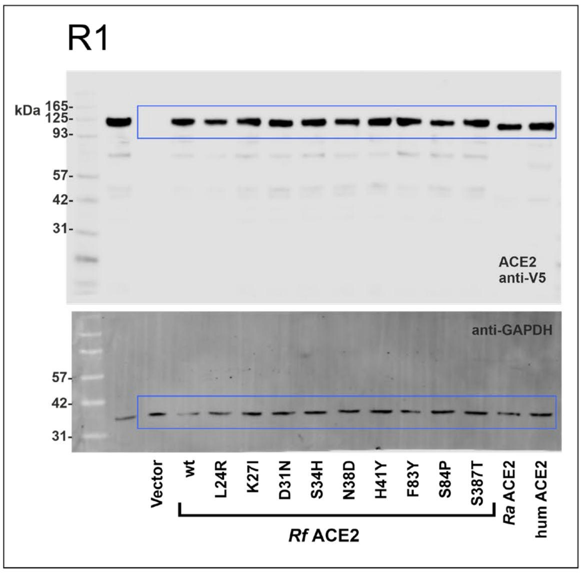

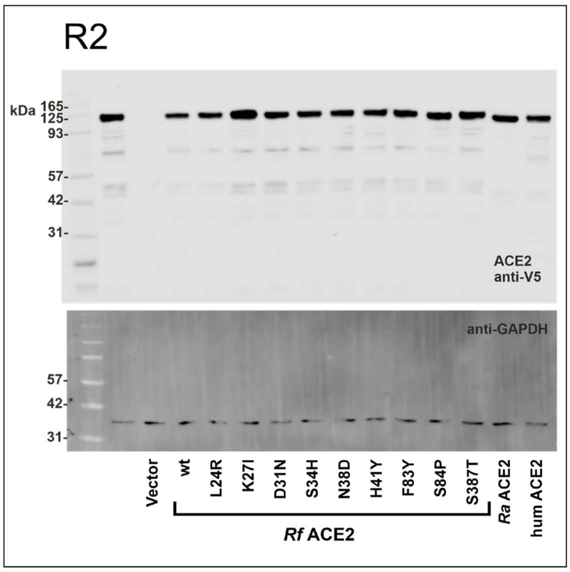

Above single membrane staining with
2 different channels of Secondary antibody

Above single membrane staining with
2 different channels of Secondary antibody

Supplementary Figure 9

Fig. S1a

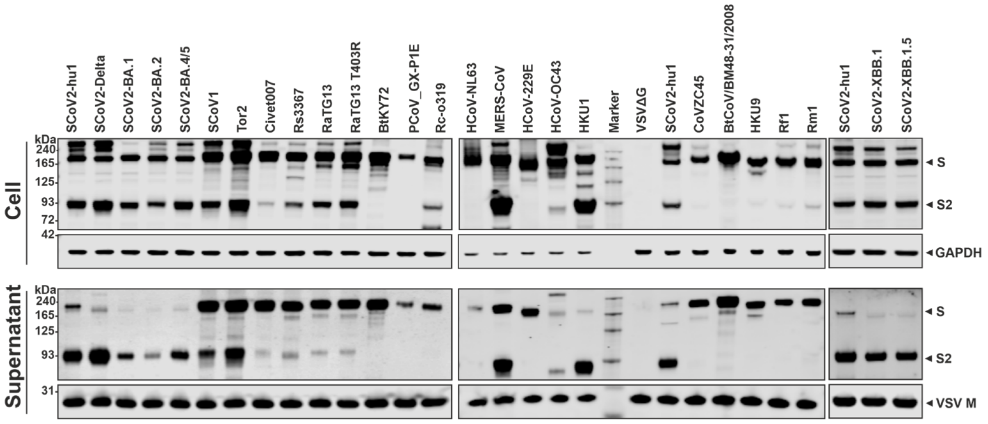

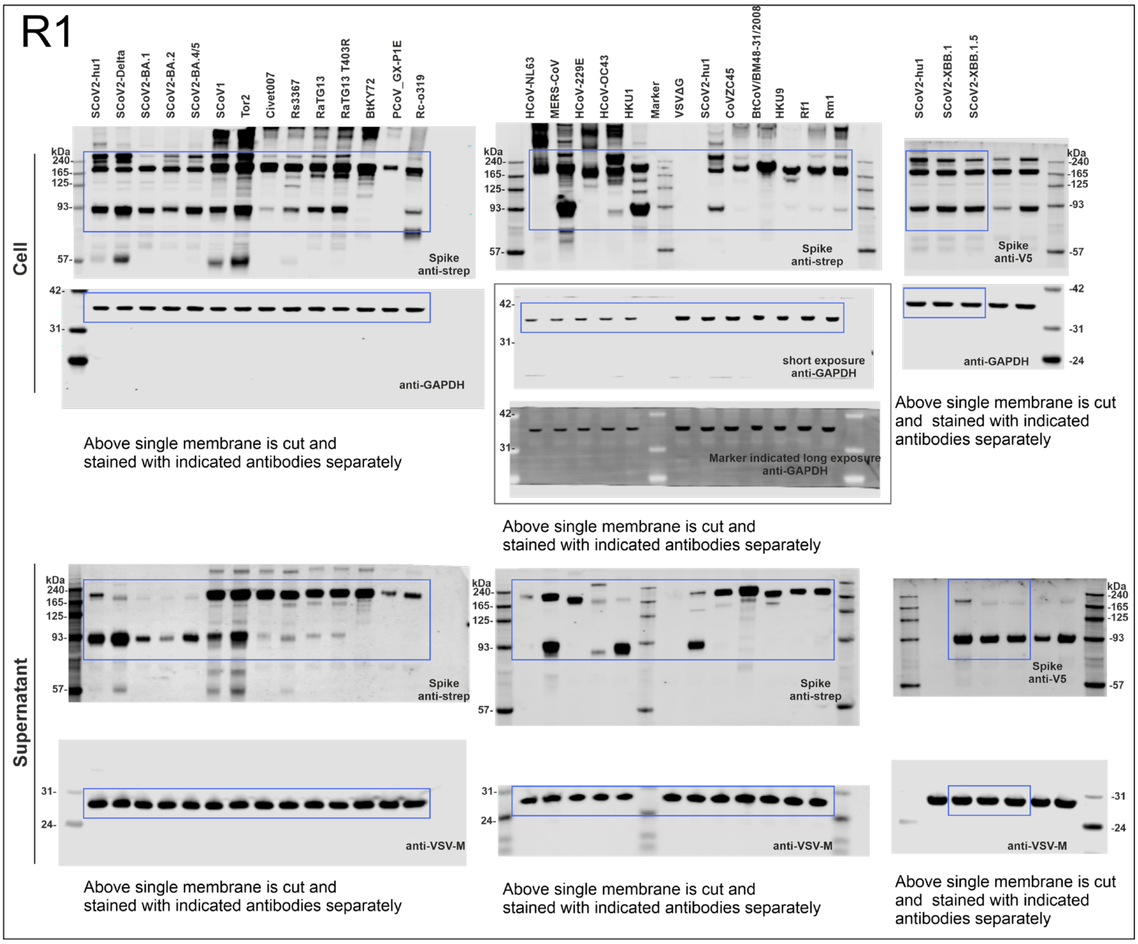

Supplementary Figure 9

Fig. S1a

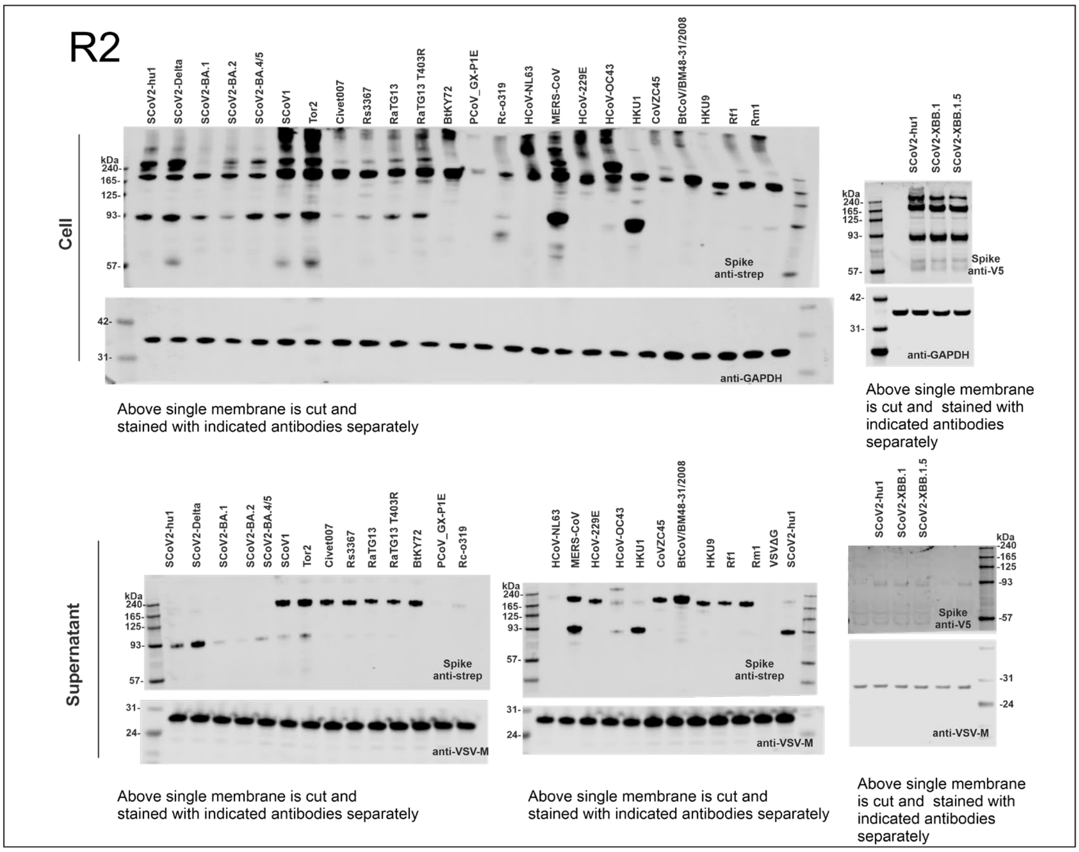

Fig.S1b

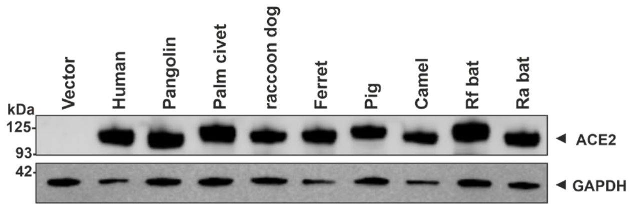

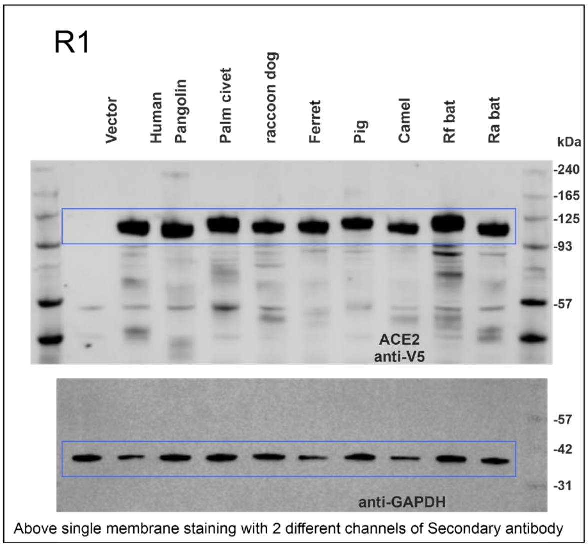

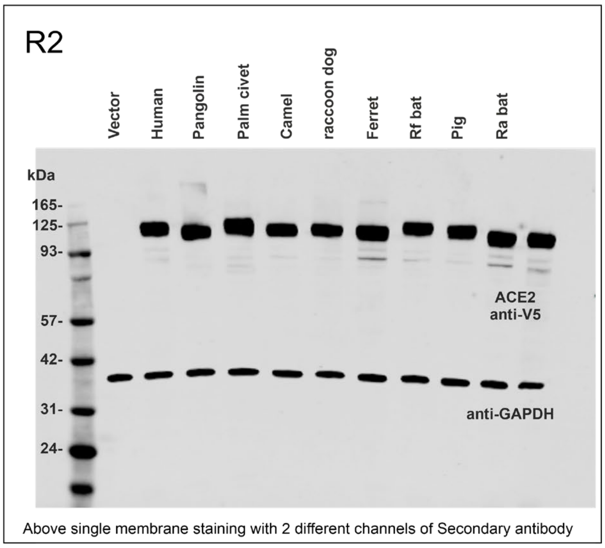

Supplementary Figure 9. Uncropped western blots. The corresponding figures and information about the creation of any figures where strips of bands are stacked vertically is provided in the figure. All bands are derived from the same membrane, except for those labeled “cell” and “supernatant” as specified in the legend to Supplementary Figure 1a.

Supplementary Table 1. Spike sequences analyzed.

Spike Name	Origin Lat. Name	Seq. identity to SCoV2 S Hu1 (%)	Accession Nr.
SARS-CoV-2 Hu1	human	100	BCN86353.1
SARS-CoV-2 Delta	human	99,21	QTW89558.1
SARS-CoV-2 Omicron BA.1	human	96,94	UFO69279.1
SARS-CoV-2 Omicron BA.2	human	97,56	UHU97100.1
SARS-CoV-2 Omicron BA.4/5	human	97,33	UPN16705.1
SARS-CoV-2 Omicron XBB.1	human	97.01	UXU98303.1
SARS-CoV-2 Omicron XBB.1.5	human	97.01	UZG29433.1
RATG13	*Rh. affinis*	97,41	QHR63300.2
GX/P1E	*M. javanica*	92,73	QIA48623.1
CoVZC45	*Rh. sinicus*	80,69	AVP78031.1
SARS-CoV-1 ShanghaiQXC2	human	78,88	AAR86775.1
Rs3367	*Rh. sinicus*	77,07	AGZ48818.1
Rc-o319	*Rh. cornutus*	76,48	BCG66627.1
SARS-CoV-1 TOR2	human	76,04	P59594.1
Civet007	*P. larvata*	75,8	AAU04646.1
Rm1	*Rh. macrotis*	74,75	ABD75332.1
Rf1	*Rh. ferrumequinum*	74,00	ABD75323.1
BtKY72	*Rh. sp.*	72,03	APO40579.1
BM48-31/2008	*Rh. blasii*	62,99	YP_003858584.1
OC43	human	37,93	AVR40344.1
HKU1	human	35,95	YP_173238.1
MERS	human	34,82	QBM11748.1
HKU9	*Rousettus sp.*	33,33	AVP25406.1
NL63	human	31,27	YP_003767.1
229E	human	31,11	NP_073551.1

Supplementary Table 2. ACE2 sequences analyzed.

ACE2 Species Name	Lat. Name	Seq. identity to human ACE2 (%)	Accession Nr.
Human	*Homo Sapiens*	100	BAB40370.1
Pangolin	*Manis javanica*	84,63	XP_017505752.1
Palm Civet	*Paguma larvata*	83,50	Q56NL1.1
Raccoon Dog	*Nyctereutes procyonoides*	83,75	ABW16956.1
Ferret	*Mustela putorius furo*	82,49	NP_001297119.1
Pig	*Sus scrofa domesticus*	81,74	XP_020935033.1
Camel	*Camelus ferrus*	83,12	XP_006194263.1
R. ferrumequinum	*Rh. Ferrumequinum*	81,23	XP_032963186.1
R. affinis	*Rh. affinis*	80,60	QMQ39229.1
