# Supplementary material for: Determinants of species-specific utilization of ACE2 by human and animal coronaviruses
Source: Commun Biol. 2023 Oct 17;6:1051. doi: 10.1038/s42003-023-05436-3 (PMC10582019; doi:10.1038/s42003-023-05436-3)
Supplement: Supplementary file 3 — Description of Additional Supplementary Files [file 42003_2023_5436_MOESM3_ESM.pdf]

Description of Additional Supplementary Files

File name: Supplementary Data 1
Description: Source data for figures and blots.

File name: Supplementary Data 2
Description: Primers used for generating plasmid constructs.
