# Supplementary material for: Determinants of species-specific utilization of ACE2 by human and animal coronaviruses
Source: Commun Biol. 2023 Oct 17;6:1051. doi: 10.1038/s42003-023-05436-3 (PMC10582019; doi:10.1038/s42003-023-05436-3)
Supplement: Supplementary file 6 — Reporting Summary [file 42003_2023_5436_MOESM6_ESM.pdf]

nature portfolio

Corresponding author(s): Frank Kirchhoff, COMMSBIO-23-1420-T

Last updated by author(s): Aug 22, 2023

Reporting Summary

Nature Portfolio wishes to improve the reproducibility of the work that we publish. This form provides structure for consistency and transparency in reporting. For further information on Nature Portfolio policies, see our Editorial Policies and the Editorial Policy Checklist.

Statistics

For all statistical analyses, confirm that the following items are present in the figure legend, table legend, main text, or Methods section.

n/a	Confirmed	
☐	☒	The exact sample size (*n*) for each experimental group/condition, given as a discrete number and unit of measurement
☐	☒	A statement on whether measurements were taken from distinct samples or whether the same sample was measured repeatedly
☐	☒	The statistical test(s) used AND whether they are one- or two-sided<br>*Only common tests should be described solely by name; describe more complex techniques in the Methods section.*
☒	☐	A description of all covariates tested
☒	☐	A description of any assumptions or corrections, such as tests of normality and adjustment for multiple comparisons
☐	☒	A full description of the statistical parameters including central tendency (e.g. means) or other basic estimates (e.g. regression coefficient) AND variation (e.g. standard deviation) or associated estimates of uncertainty (e.g. confidence intervals)
☐	☒	For null hypothesis testing, the test statistic (e.g. *F*, *t*, *r*) with confidence intervals, effect sizes, degrees of freedom and *P* value noted<br>*Give P values as exact values whenever suitable.*
☒	☐	For Bayesian analysis, information on the choice of priors and Markov chain Monte Carlo settings
☒	☐	For hierarchical and complex designs, identification of the appropriate level for tests and full reporting of outcomes
☒	☐	Estimates of effect sizes (e.g. Cohen's *d*, Pearson's *r*), indicating how they were calculated

Our web collection on statistics for biologists contains articles on many of the points above.

Software and code

Policy information about availability of computer code

Data collection	Odyssey LI-COR Image Studio Lite Version 5.2, Cytation 3 microplate reader (BioTek Instruments)
Data analysis	BioTek Gen5, GraphPad Prism Version 9.4.1, Corel DRAW 2021.5,Odyssey LI-COR Image Studio Lite Version 5.2, Fiji 1.53, ClustalW in msa (1.18.0), ggplot2 (3.3.5), ggseqlogo (0.1), Amsterdam Modeling Suite 2020, VMD 1.9.3, R (3.6.1)

For manuscripts utilizing custom algorithms or software that are central to the research but not yet described in published literature, software must be made available to editors and reviewers. We strongly encourage code deposition in a community repository (e.g. GitHub). See the Nature Portfolio guidelines for submitting code & software for further information.

Data

Policy information about availability of data

All manuscripts must include a data availability statement. This statement should provide the following information, where applicable:

- Accession codes, unique identifiers, or web links for publicly available datasets
- A description of any restrictions on data availability
- For clinical datasets or third party data, please ensure that the statement adheres to our policy

The datasets generated during and/or analyzed during the current study are available from the corresponding authors on request. All Source data are provided in the Supplementary Information/Source Data file. The SARS-CoV-2 S structure used in this study is available in the Protein Data Bank (PDB) under accession code 3KBH (https://www.rcsb.org/structure/3KBH); Primary coronavirus and ACE2 sequences are available from the National Center for Biotechnology and Information

(NCBI): https://www.ncbi.nlm.nih.gov/nuccore/(BCN86353.1, QTW89558.1, UFO69279.1, UHU97100.1, UPN16705.1, QHR63300.2, QIA48632.1, AVP78031.1, AAR86775.1, AGZ48818.1, BCG66627.1, P59594.1, AAU04646.1, ABD75332.1, ABD75323.1, APO40579.1, YP_003858584.1, AVR40344.1, YP_173238.1, QBM11748.1, AVP25406.1, YP_003767.1, NP_073551.1, BAB40370.1, XP_017505752.1, XP_017505752.1, ABW16956.1, NP_001297119.1, XP_020935033.1, XP_006194263.1, XP_032963186.1, QMQ39229.1)

Research involving human participants, their data, or biological material

Policy information about studies with human participants or human data. See also policy information about sex, gender (identity/presentation), and sexual orientation and race, ethnicity and racism.

Reporting on sex and gender	Donor 1, female, AZ-BNT-BNT<br>Donor 4, male, AZ-BNT-BNT<br>Donor 7, male, AZ-BNT-BNT<br>Donor 8, female, AZ-BNT-BNT<br>Donor 9, male, AZ-BNT-BNT<br>Donor 33, male, BNT-BNT-BNT<br>Donor 34, female, BNT-BNT-BNT<br>Donor 36, female, BNT-BNT-BNT<br>Donor 37, male, BNT-BNT-BNT<br>Donor 38, male, BNT-BNT-BNT<br>It has no relevance for the current study.
Reporting on race, ethnicity, or other socially relevant groupings	No such information was collected and is of no relevance for the sera used in this study.
Population characteristics	Donor 1, 1995<br>Donor 4, 1975<br>Donor 7, 1995<br>Donor 8, 1991<br>Donor 9, 1995<br>Donor 33, 1961<br>Donor 34, 1999<br>Donor 36, 1995<br>Donor 37, 1985<br>Donor 38, 1995<br>It has no relevance for the current study.
Recruitment	Ulm University Medical Center Employees who were vaccinated three times,had no indication of previous SARS-CoV-2 infection and expressed interest in participating were included the present study.
Ethics oversight	Ethic Committee of Ulm University (vote 99/21– FSt/Sta).

Note that full information on the approval of the study protocol must also be provided in the manuscript.

Field-specific reporting

Please select the one below that is the best fit for your research. If you are not sure, read the appropriate sections before making your selection.

☒ Life sciences ☐ Behavioural & social sciences ☐ Ecological, evolutionary & environmental sciences

For a reference copy of the document with all sections, see nature.com/documents/nr-reporting-summary-flat.pdf

Life sciences study design

All studies must disclose on these points even when the disclosure is negative.

Sample size	Sample sizes were chosen according to established protocols and not statistically assessed. Experiments were replicated, as indicated in the Statistics and Reproducability section or the corresponding figure legends usually at least three times independently to verify the results.
Data exclusions	No data were excluded.
Replication	The number of independent replicates is indicated in the respective figure legends. All attempts at replication yielded similar results. N/A
Randomization	Randomization was not applicable for this study, as no human trials or cohort studies were performed or samples assigned to experimental groups.
Blinding	Blinding was not applicable for results that were quantified using machines and no human manual quantification was involved.

Reporting for specific materials, systems and methods

We require information from authors about some types of materials, experimental systems and methods used in many studies. Here, indicate whether each material, system or method listed is relevant to your study. If you are not sure if a list item applies to your research, read the appropriate section before selecting a response.

Materials & experimental systems

n/a	Involved in the study	
☐	☒	Antibodies
☐	☒	Eukaryotic cell lines
☒	☐	Palaeontology and archaeology
☒	☐	Animals and other organisms
☒	☐	Clinical data
☒	☐	Dual use research of concern
☒	☐	Plants

Methods

n/a	Involved in the study	
☒	☐	ChIP-seq
☒	☐	Flow cytometry
☒	☐	MRI-based neuroimaging

Antibodies

Antibodies used

strep, Thermo Fisher Scientific, #PA5-119611
V5-tag, Cell Signalling, #13202
VSV-M, Absolute Antibody, 23H12, #Ab01404-2.0
GAPDH, BioLegend, #631401

IRDye® 800CW Goat anti-Rat IgG Secondary Antibody, Li-CORE, Cat#926-32219, C91211-09, dilution (1:10000)
IRDye® 680CW Goat anti-Mouse IgG Secondary Antibody,Li-CORE, Cat#926-68070, lot C90910-21, dilution (1:10000)

Validation

https://www.licor.com/bio/reagents/irdye-800cw-goat-anti-rabbit-igg-secondary-antibody

Eukaryotic cell lines

Policy information about cell lines and Sex and Gender in Research

Cell line source(s)

Human HEK293T cells, ATCC Cat# CRL-3216
Mouse I1-Hybridoma cells, ATCC Cat#CRL2700

Authentication

The cell lines were authenticated by ATCC and not validated further in our laboratory.

Mycoplasma contamination

Cells were tested routinely to be free of mycoplasma using a PCR based test.

Commonly misidentified lines
(See ICLAC register)

no commonly misidentified cell lines were used in this study.
